# Current status and future developments in dental implantology – a narrative review

**DOI:** 10.1515/iss-2025-0020

**Published:** 2025-09-18

**Authors:** Constanze Friedrich, Constantin Graw, Juliane Kröplin

**Affiliations:** Department of Oral and Maxillofacial Surgery, University Medical Centre Rostock, Rostock, Germany

**Keywords:** dental implants, navigation, digital surgical planning, robotics, automation

## Abstract

**Introduction:**

The vast majority of people are affected by tooth loss in the course of their lives. Dental implants are used to anchor prosthetic teeth and represent the gold standard for replacing missing teeth. As the preservation of chewing function and esthetics has a significant influence on the quality of life, the constant further development of techniques and materials is a central issue in oral and maxillofacial surgery. This narrative review article aims to evaluate the historical development, current status, and innovative methods of dental implants.

**Content:**

A selective literature search based on Pubmed was conducted, focusing primarily on literature from the years 2023 to 2025. Keywords used in the search were “dental implants” in combination with “navigation” and “virtual surgical planning.”

**Summary:**

The causes of tooth loss, the anatomical conditions of the jaw, and possible treatment approaches for oral rehabilitation are diverse and require individual consideration depending on the individual patient situation. In any case, the use of materials and geometries supporting the osseointegration of the implants, the optimal positioning of the implants in the jawbone, and surgery procedures that are as atraumatic as possible are fundamental to successful implantation. In recent years, digital and technical innovations have changed the way treatment is planned and performed. The shift toward an increasingly digital workflow has resulted in more efficient planning and improved precision in implant positioning. This is associated with improved clinical results and more satisfied patients.

**Outlook:**

In this study, the field of dental implantology is reviewed and future opportunities and challenges are identified. Despite the fact that this will result in new obstacles, dental implantation can become increasingly precise and efficient thanks to the development of digital technologies such as Augmented Reality-Assisted Navigation and autonomous dental implant robots.

## Introduction

Tooth loss and the need for prosthetic restauration affect large parts of our society. According to the latest health report from the Robert Koch Institute, only 12.6 % of men and 14.4 % of women in Germany have full dentition by the age of 35–44 [1]. Impaired chewing function due to tooth loss can result in reduced oral health. In these cases, a dental prosthesis is indicated in order to restore sufficient functionality of the masticatory organ or to prevent its impairment. The treatment can be carried out using bridges or removable partial dentures, for example, while completely edentulous jaws can be treated with a complete denture. However, dental implants are the gold standard for replacing missing natural teeth [2]. They are positioned in the alveolar ridge and serve as anchors for dental prostheses in the form of crowns, bridges, or dentures. In fully or partially edentulous jaws, dental implants are indicated and show good clinical results. Over observation periods of 8–10 years, survival rates of over 95 % can be found, depending on the literature [3], 4].

The first descriptions of dental implants date back to the Majas around 600 AD. X-ray images from the 1970s of the lower jaws of Majas showed shells surrounded by compact bone. In the 20th century, the development of dental implants gained significant momentum, with multiple innovations in shape, material, and surface texture [5].

The most commonly used implants today are screw implants, which imitate the shape of the natural tooth root. Depending on the manufacturer and model, they can be one-piece or two-piece. There are also various surgical protocols, which can basically be divided into three different approaches: one-stage procedure, two-stage procedure, and immediate implant loading. In a two-stage procedure, only the implant body is initially inserted underneath the mucosa. After waiting for the bone to heal (usually 2–6 months), the implant is exposed in a second surgical procedure and an abutment is inserted to shape the soft tissue. In a single-stage procedure or protocols with immediate loading, this second surgical procedure is not necessary. The choice of protocol depends on multiple factors, including both the patient’s individual situation and the practitioner’s preferences [6].

For special cases, such as patients with cleft lip and palate, patients after maxillectomy due to tumors or failed implantation in the upper jaw, special zygomatic implants are available in addition to conventional implants. These can be inserted into the zygomatic bones if there is insufficient bone in the upper jaw to anchor dentures [7].

## Methods

A literature search was conducted in PubMed, focusing on publications from the years 2023 to 2025. The search mainly targeted open access articles covering current trends and new evidence-based strategies using the search terms “dental implants” in combination with “virtual surgical planning” and “navigation.” Given the selective nature of the search, no strict criteria were applied, which allowed for a flexible literature collection. The literature included comprised reviews, prospective cohort studies, narrative reviews, textbooks, retrospective studies, and systematic reviews. A total of 23 studies were included. Although the focus was primarily on innovations, some older articles were also included to provide basic information and increase the comprehensibility of the subject area for a broader audience.

## Dental implants – shape, materials, surface

During normal chewing, large forces of up to 100 N can occur, which are distributed via the teeth to the periodontium and the jawbone. During the healing process, no physiological periodontium forms around dental implants. A sufficient connection between the implant and the bone must, therefore, be established after implantation, so that the chewing forces can be transferred to the bone via the implants without the implants becoming loose. This process called osseointegration was discovered by chance in 1952 by Dr. P. Brånemark when he inserted titanium chambers into the femur of rabbits and found that they could no longer be removed after a while because they had fused with the bone. Brånemark defined the term osseointegration as “a direct structural and functional connection between ordered, living bone, and the surface of a load carrying implant” [5]. The osseointegration of dental implants usually takes 3–6 months. Multiple factors influence the likelihood of successful osseointegration, such as optimal surgical techniques and factors provided by the organism into which the implant is inserted. The material and the surface of the implants are also important factors that significantly influence osseointegration. By using suitable materials, the time until the implants can be loaded can be shortened and the proportion of the implant surface that is in direct bone-to-implant contact can be increased, which in turn increases the load-bearing capacity of the implants.

Different materials have been used for dental implants over the last 6–7 decades. After models made of cobalt and chromium or stainless steel, titanium implants in particular have become increasingly widespread. Titanium has been used for dental implants since the mid-1960s and has shown excellent clinical results, partly due to its high biocompatibility and mechanical properties. Nonmetallic alternatives, which have also proven themselves clinically, are implants made of zirconium oxide or aluminum oxide ceramics and their alloys. Unlike titanium implants, zirconium implants offer better aesthetics. They also feature low plaque adhesion and mechanical properties comparable to those of titanium implants. Over short observation periods, they show survival rates similar to titanium implants; results on long-term survival rates are still pending. The materials mentioned are only a selection; multiple metallic and nonmetallic materials are being researched for use in dental implants. In addition to the material from which the implants are made, osseointegration can also be improved by modifying the implant surface. Various physical and chemical processes exist for activating the surface, as well as coating with bioactive substances such as hydroxyapatite. Suitable coating materials can improve the osseointegration of implants by improving direct bone-to-implant contact. The shape of implants has also been regularly developed further to enable optimal healing and load-stable osseointegration. While some very unusual geometries such as blade implants have been designed over time, screw-shaped implants, whose shape is similar to that of the natural tooth root, are now the most popular [5], 6].

## Risk factors for implant failure

Besides these implant-related factors, the long-term success of dental implants is strongly influenced by individual patient-related risk factors. Poor glycemic control in patients with diabetes mellitus has been shown to significantly impair both wound healing and osseointegration, potentially leading to higher implant failure rates. Similarly, obesity can increase the risk of peri-implantitis due to elevated systemic inflammation and changes in bone metabolism. Elevated C-reactive protein (CRP) levels can be an indicator of chronic inflammation, which may further complicate healing processes following implantation [8].

Smoking is another major risk factor that negatively affects vascularization, delays tissue regeneration, increases susceptibility to infection, and exacerbates inflammation in the oral cavity. In patients with osteoporosis or those undergoing bisphosphonate therapy, impaired bone remodeling may hinder the osseointegration of implants. Additional challenges arise in patients with immunosuppressive conditions, malnutrition – particularly vitamin D deficiency – or those who have received radiotherapy in the head and neck region, all of which can reduce healing capacity and compromise implant prognosis.

Another often underestimated risk is bruxism, which may cause mechanical overload and micromovements, thereby jeopardizing the primary and secondary stability of implants [9], 10].

To ensure optimal outcomes, early identification and management of these risk factors is essential. Preoperative screening, interdisciplinary collaboration, and patient education form the basis for a tailored treatment plan that minimizes complications and supports long-term implant survival.

## Digital technologies in modern implantology

Technological advancements are fundamentally transforming the field of dental implantology. Digital tools now support the entire treatment process – from diagnostics and planning to surgical execution and patient communication.

### Virtual planning and navigation

Due to the lack of alternatives, dental implants were planned in the past mainly by means of clinical examination and two-dimensional imaging using dental films and orthopantomograms. Two-dimensional imaging of three-dimensional structures results in diagnostic uncertainty. This can lead to problems during and after implant placement, particularly when assessing the horizontal bone volume.

Modern implant planning begins with three-dimensional imaging. Cone Beam Computed Tomography (CBCT) has become a standard diagnostic tool, offering high-resolution insights into the patient’s bone morphology, nerve pathways, and sinus and tooth positions [11]. Using CAD/CAM software, clinicians can virtually position implants based on anatomical and prosthetic criteria. In the concept of prosthetic-driven implant placement, the implants are planned based on the desired future denture. This allows for optimal selection and positioning of the implants based on aesthetic and mechanical requirements, resulting in a beneficial emergence profile and longevity due to optimal distribution of the forces acting on the implant [12]. These plans can be translated into surgical guides, allowing for template-based procedures with high reproducibility [13].

More dynamic still are navigation systems, which provide real-time guidance during drilling. Sensors track the position of the surgical handpiece and project the planned trajectory on-screen, enhancing precision and reducing human error [13], 14].

### Robotics and automation

Dental implant robots are computer-controlled systems designed to assist or autonomously perform parts of the surgical implant procedure based on a preoperative digital treatment plan. These systems typically use 3D imaging data and planning software to guide the drill path. The advent of robot-assisted implant placement marks a major leap forward in procedural accuracy and reproducibility. Robotic systems allow for automated, preprogrammed osteotomies, executed with submillimeter precision. These systems minimize deviation from planned implant positions and standardize surgical outcomes [15], 16].

*In vitro* and clinical studies confirm that robotic surgery can outperform both freehand and static guide techniques in terms of positional accuracy, especially in challenging anatomic situations, such as posterior regions or narrow ridges [17], 18].

### Artificial intelligence in diagnostic imaging and treatment planning

Artificial intelligence (AI) is rapidly becoming an integral part of modern dental implantology. One of its primary applications lies in diagnostic imaging, particularly in identifying anatomical structures and pathological changes in CBCT scans and panoramic radiographs. AI models have shown high sensitivity and specificity in detecting peri-implant bone loss and early signs of peri-implantitis [9], 10], 19].

In addition, AI systems are increasingly used for the automated segmentation of hard and soft tissues from three-dimensional imaging data. This not only reduces manual workload but also minimizes interoperator variability, leading to more standardized diagnostics [9], 19]. Further developments include tools capable of automatically identifying specific implant systems from radiographic images. These systems, trained on extensive annotated datasets, support clinicians in retrospective case analysis and enhance diagnostic consistency across diverse clinical settings [19].

AI also plays a growing role in personalized treatment planning. Predictive algorithms trained on large-scale clinical datasets are capable of analyzing complex interactions between bone quality, systemic risk factors, and prosthetic load. These systems can generate evidence-based recommendations for implant dimensions, positions, and configurations. Increasingly, such AI-driven tools are being integrated into digital planning workflows, enabling more individualized, data-driven treatment strategies and potentially improving long-term clinical outcomes [11], 13], 19].

The integration of 3D printing further strengthens the connection between AI-supported planning and clinical execution. High-resolution printers allow for the rapid fabrication of patient-specific surgical guides, anatomical models, and planning prototypes directly from files derived from digital planning software. This facilitates enhanced preoperative visualization and contributes to the accuracy and predictability of surgical procedures [13], 18].

### Virtual, augmented, and mixed reality in modern implant surgery

Within the broader context of digital visualization technologies, it is essential to distinguish between Virtual Reality (VR), Augmented Reality (AR), and Mixed Reality (MR), as each offers distinct applications in oral and maxillofacial surgery [20]. Virtual Reality refers to fully immersive, computer-generated environments that are primarily used in education and surgical training. VR simulators enable both students and experienced practitioners to perform implant procedures in a safe, simulated setting. These systems have been shown to improve spatial awareness, hand-eye coordination, and procedural confidence [21]. In addition to training, VR is also being used in patient communication, allowing clinicians to present interactive 3D models of the patient’s anatomy, thereby improving understanding, transparency, and informed consent [21].

Augmented Reality, by contrast, overlays digital information onto the physical world, often through head-mounted displays or tablet-based systems. In the context of implant surgery, AR enables the real-time visualization of anatomical landmarks, planned implant trajectories, and critical structures such as the inferior alveolar nerve. This integration enhances surgical orientation and precision, particularly in anatomically challenging regions [14], 22].

Mixed Reality combines elements of both VR and AR, allowing virtual 3D models to be interactively integrated into the clinical environment. This technology enables clinicians to view, manipulate, and simulate patient-specific anatomical structures in real time [23]. In combination with robotic systems and dynamic navigation platforms, these technologies are paving the way for splintless implant placement protocols [23], in which implants can be placed with high accuracy without the need for physical surgical guides [15], [16], [17, 21], 22]. This reduces preparation time, material use, and increases intraoperative flexibility [13], 18].

In summary, while VR primarily enhances education and patient communication [21], AR contributes to greater surgical precision [14], 22], and MR fosters interactive, collaborative planning [15], 22]. A clear differentiation of these technologies underscores their complementary roles and highlights their respective contributions to the advancement of contemporary digital implantology [21].

Despite these promising developments, several challenges remain. High initial costs for hardware, software, and training, as well as the complexity of integrating digital systems into existing clinical workflows, may limit widespread adoption. Addressing these barriers – through streamlined software interfaces, interoperable systems, and AI-driven automation – will be essential to ensure that digital technologies are accessible, efficient, and ultimately beneficial to a broader range of clinicians and patients [13], 14], 21].

## Summary and conclusion

Dental implantology has undergone a profound transformation in recent years, evolving from a primarily mechanical discipline into a highly digitalized, biologically informed specialty. This narrative review has highlighted how the success of implant therapy is shaped by a combination of material science, surgical technique, anatomical conditions, and increasingly, digital technologies.

Implants today benefit from advances in biomaterials and surface modifications that promote faster and stronger osseointegration. The choice of surgical protocol – whether conventional, immediate, or staged – must be tailored to each patient’s individual risk profile, systemic health, and anatomical constraints.

Digital tools now play a central role in diagnosis, planning, and execution. CBCT imaging, CAD/CAM planning, dynamic navigation, and augmented reality are streamlining workflows and enhancing surgical accuracy. Robotic systems are emerging as powerful tools for consistent and precise implant placement. Meanwhile, artificial intelligence supports clinicians in diagnostics and decision-making, while virtual reality improves training environments and patient communication.

Despite these advances, challenges remain. Systemic risk factors such as diabetes, smoking, or osteoporosis continue to impact clinical outcomes. Additionally, the integration of advanced technologies requires investment in training and infrastructure.

Nonetheless, the overall trajectory of the field points toward fully integrated digital workflows that combine biological compatibility, technological precision, and data-driven planning. Future innovations are expected to focus on smart implant surfaces, AI-supported diagnostics, and autonomous surgical systems – offering patients safer, more personalized, and more efficient care.
